# Clustering of pain and its associations with health in people aged 50 years and older: cross-sectional results from the North Staffordshire Osteoarthritis Project

**DOI:** 10.1136/bmjopen-2015-008389

**Published:** 2015-11-09

**Authors:** R J Lacey, V Y Strauss, T Rathod, J Belcher, P R Croft, B Natvig, R Wilkie, J McBeth

**Affiliations:** 1 Arthritis Research UK Primary Care Centre, Research Institute for Primary Care & Health Sciences, Keele University, Keele, Staffordshire, UK; 2 Centre for Statistics in Medicine, University of Oxford, Botnar Research Centre, Oxford, UK; 3 School of Computing and Mathematics, Keele University, Keele, Staffordshire, UK; 4 Department of General Practice, Institute of Health and Society, University of Oslo, Oslo, Norway

**Keywords:** EPIDEMIOLOGY, PRIMARY CARE, RHEUMATOLOGY

## Abstract

**Objective:**

Most pain in patients aged ≥50 years affects multiple sites and yet the predominant mode of presentation is single-site syndromes. The aim of this study was to investigate if pain sites form clusters in this population and if any such clusters are associated with health factors other than pain.

**Setting:**

Six general practices in North Staffordshire, UK.

**Design:**

Cross-sectional, postal questionnaire, study.

**Participants:**

Community-dwelling adults aged ≥50 years registered at the general practices.

**Main outcomes measures:**

Number of pain sites was measured by asking participants to shade sites of pain lasting ≥1 day in the past 4 weeks on a blank body manikin. Health factors measured included anxiety and depression (Hospital and Anxiety Depression Scale), cognitive complaint (Sickness Impact Profile) and sleep. Pain site clustering was investigated using latent class analysis. Association of clusters with health factors, adjusted for age, sex, body mass index and morbidities, was analysed using multinomial regression models.

**Results:**

13 986 participants (adjusted response 70.6%) completed a questionnaire, of whom 12 408 provided complete pain data. Four clusters of participants were identified: (1) low number of pain sites (36.6%), (2) medium number of sites with no back pain (31.5%), (3) medium number of sites with back pain (17.9%) and (4) high number of sites (14.1%). Compared to Cluster 1, other clusters were associated with poor health. The strongest associations (relative risk ratios, 95% CI) were with Cluster 4: depression (per unit change in score) 1.11 (1.08 to 1.14); cognitive complaint 2.60 (2.09 to 3.24); non-restorative sleep 4.60 (3.50 to 6.05).

**Conclusions:**

These results indicate that in a general population aged ≥50 years, pain forms four clusters shaped by two dimensions—number of pain sites (low, medium, high) and, within the medium cluster, the absence or presence of back pain. The usefulness of primary care treatment approaches based on this simple classification should be investigated.

**Strengths and limitations of this study**

- This was a large population study of community-dwelling adults aged ≥50 years, 48% of whom were aged ≥70 years.
- There was a high response to the study (70.6%).
- We measured several potential confounders of the relationship between pain clusters and health factors that are common in those with multisite pain and of age ≥50 years.
- The data are cross-sectional, so we cannot specify the direction of the associations.
- We did not assess physical health outcomes or pain intensity at each pain site.

## Introduction

Multisite pain is common in adults aged ≥50 years,^1–3^ with 40% of persons aged ≥65 years reporting pain at ≥2 sites.^4^ Despite the common occurrence of multisite pain, the clinical presentation, assessment and treatment of pain has tended to focus on single sites of pain.^5^ For example, in a UK consultation database of 12 general practices, 86% of musculoskeletal primary care consultations were for pain at a single body region compared with 8% defined as a generalised/widespread problem.^6^ This may reflect the prioritisation of pain problems by patients and general practitioners, both of whom are aware of the limited time available in a routine primary care consultation.^5^

The presence of pain occurring at site(s) additional to the one presented increases the likelihood and severity of disability in people aged ≥50 years.^2^ Indeed, the extent of pain has a strong dose–response relationship with mental and physical health outcomes in this age group, including sleep disturbances,^7^ falls,^8^ lower extremity dysfunction^4^ and cognitive complaint.^9^ In separate analyses, Leveille *et al*^8^ demonstrated an association between falls and number of pain sites, and falls and location of pain. However, these studies did not account for the possibility that distinct patterns of multisite pain may exist, with respect to the number and location of pain sites taken into account simultaneously, which may be of practical value in the management of pain in people aged 50 years and above. Evidence from an existing study of patterns of multisite pain suggests that patterns may differ in adults aged ≥50 compared with those aged ≤50 years, although the study focus was not the older age group.^10^ As adults over 50 years live longer, the impact of multisite pain on individuals and health services is likely to increase. Identification of specific patterns of multisite pain in people aged ≥50 years, which may also be differentially associated with health outcomes, may provide a basis for developing improved clinical assessment and targeted treatment of multisite pain in this age group of primary care patients consulting with apparent single site pain.

The aim of our study was to determine, in persons aged ≥50 years, if pain sites form clearly defined clusters. Secondary analyses tested whether pain clusters were associated with clinically important health factors known to be linked with pain in people aged ≥50 years (sleep, anxiety, depression and cognitive complaint).

## Methods

For purposes of clarity and brevity, we will refer to the study population of persons aged ≥50 years as ‘older people’ in the rest of this paper.

This study drew on information from the North Staffordshire Osteoarthritis Project (NorStOP). NorStOP was a series of three community-based prospective cohort studies of joint pain and general health in the registered older adult populations of general practices in North Staffordshire, UK. The current study used baseline data, collected between 2002 and 2003, from two cohorts of NorStOP. Details of the NorStOP methods and sample size have been published previously.^11–13^ Older adults were defined as aged ≥50 years for NorStOP, since age-related rates of chronic disease including osteoarthritis start to rise dramatically, and general practitioners’ labelling of joint pain starts to reflect the probability that the reason for joint pain is likely to be osteoarthritis, from age 50 upwards.^14^ Consent to use the data collected in the questionnaires was implied through the returning of the questionnaires to the research centre.^9^ ^14^ Approval for the study was granted by the North Staffordshire Research Ethics Committee (REC reference numbers 1351 and 1430).

### Study population

**Figure 1:**
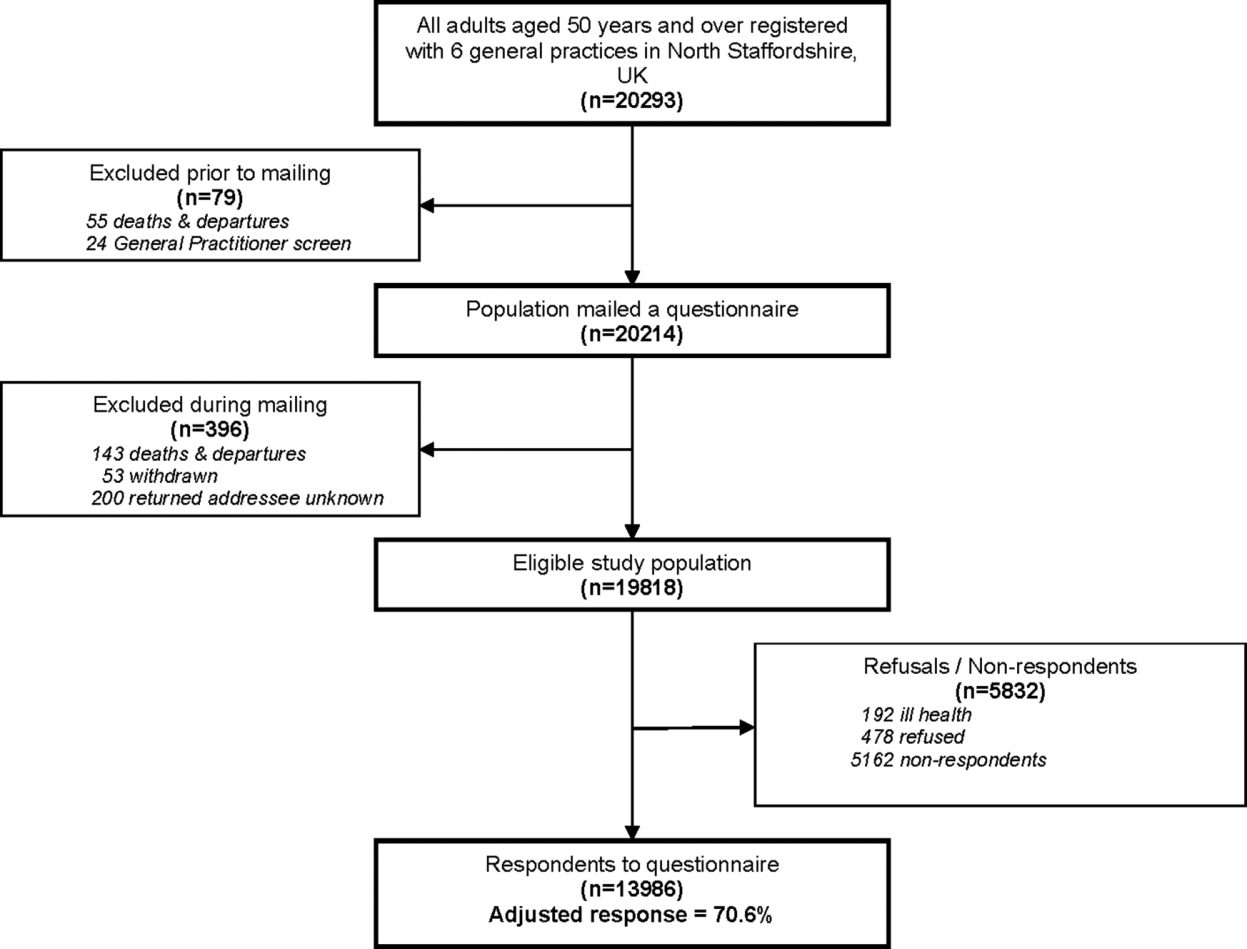
*Flowchart of numbers of individuals at each stage of the* study: Flowchart showing the sampling frame for the study, numbers of individuals excluded and the reasons for their exclusion at all stages of mailing, and response to the study.

The sampling frame for the current study was all adults aged ≥50 years registered with six general practices (n=20 293; figure 1), which are part of Primary Care Clinical Research Network: West Midlands (http://www.crn.nihr.ac.uk/west-midlands/). In the UK, about 98% of the British population are registered with a general practitioner (GP),^15^ regardless of whether they seek treatment or not; hence the registers provide a convenient means of obtaining a representative sample of the general population in a locality. Following a screen by GPs, 79 people were excluded, for example, due to severe psychiatric or terminal illness. A total of 20 214 self-complete questionnaires were mailed with a letter from the general practice and a study information leaflet. The questionnaire included items on pain, demographic factors, health behaviours, psychological factors and morbidities. During mailing, 396 people were excluded (deaths, departures from the practices and addressees unknown) giving an eligible study population of 19 818. To maximise response to the survey, reminders were sent to non-responders 2 and 4 weeks after the initial questionnaire.

### Study questionnaire

#### Pain ascertainment

Participants were asked if, in the past 4 weeks, they had had pain that had lasted for 1 day or longer in any part of their body. Those answering positively to this question were asked to shade the site of their pain(s) on a blank body manikin with front and back views. For all questionnaires returned, completed manikins were scored using a transparent template which was placed over each completed manikin and the number of painful sites for each participant was recorded.^11^ This method has been used widely in population studies of pain to measure pain location,^2^ ^3^ ^9^ ^11^ ^14^ has been shown to have good reliability,^16^ ^17^ and to give a similar prevalence of pain to written questions.^18^ The data from the scored manikins was used to identify those participants reporting pain or no pain in each of the following 16 sites: head, neck, chest, abdomen, shoulder, elbow, forearm, hand, spine, upper back, lower back, buttock, thigh, knee, calf and shin, foot.

#### Self-reported health measures

1. Anxiety and depression were measured using the Hospital Anxiety and Depression Scale (HADS).^19^ The 14-item HADS has two subscales, anxiety and depression, each comprising seven items scored 0–3, with a total score range from 0 to 21; if one item is unanswered in either the anxiety or depression subscale, it is substituted with the mean of the other six values that are present.^19^ The HADS has been used widely in population studies of pain to measure anxiety and depression.^2^ ^9^ ^13^ ^14^ A review of the validity of the HADS concluded it performed well in screening for the separate dimensions of anxiety and depression, and caseness of anxiety disorders and depression, in non-psychiatric hospital clinic, general practice and psychiatric patients, and the general population.^20^ The review found acceptable internal consistencies for anxiety (Cronbach's α 0.68–0.93) and depression (0.67–0.90), and evidence for a two-factor structure,^20^ although some correlation between the two subscales existed.^20^ ^21^
2. Cognitive complaint (the perception of cognitive impairment) was measured using the Alertness Behaviour Subscale (ABS), one of the 12 domains in the Sickness Impact Profile (SIP).^22^ The ABS contains 10 items describing alertness and ability to concentrate, to which respondents answer ‘yes’ or ‘no’; higher scores represent worse cognitive complaint. The positive answers are weighted, summed and converted to a percentage.^9^ ^22^ A previous study has shown that responders aged ≥50 years with no cognitive complaint, that is, a score of 0 on the ABS, represent over 50% of this age population.^9^ Since our aim was to investigate whether there were overall differences in cognitive complaint between the clusters, rather than classify individuals on the ABS, we took a statistical approach of categorising all positive ABS scores (1–100) into approximately equal size groups as in the previous study,^9^ to represent mild, moderate, severe and very severe cognitive complaint. There is generally good evidence for the validity of the SIP in rheumatology studies.^23^ Although the reliability of some subscales of the SIP are reported to have problems due to ceiling effects,^23^ the reliability of the ABS has been shown to be acceptable (Cronbach's α 0.76) in musculoskeletal patients.^24^
3. Sleep problems were measured using four questions regarding symptoms of poor sleep quality developed by Jenkins *et al*:^25^ “Over the past 4 weeks, did you: (a) Have trouble falling asleep? (b) Wake up several times per night? (c) Have trouble staying asleep? (d) Wake up after your usual amount of sleep feeling tired and worn out?” Each question had three possible response categories: Not at all, On some nights and On most nights. This method has been used previously in several studies.^14^ ^26–28^ To examine the overall differences in the four sleep problems between the clusters, rather than classify individuals according to insomnia, each item was analysed individually as in previous studies.^13^ ^26^

#### Potential confounding factors

Body mass index (BMI) was calculated from self-reported height and weight (weight (kg)/height (m)^2^). Morbidities commonly associated with older age were counted (from 0 to 6) by asking participants if they suffered from any of the following six health problems: chest problems, heart problems, deafness, eyesight problems (excluding the need for glasses), diabetes or raised blood pressure.^12^ These morbidities were used to investigate differences in health problems between clusters, rather than the specific nature of those differences; these morbidities represent one of a number of possible approaches to measuring comorbidity in groups and have been used in previous studies.^3^ ^13^ ^29^

### Statistical analysis

The analysis included participants who provided complete pain data, defined as either ‘yes’ to pain in past 4 weeks question and shading on the manikin, or ‘no’ to pain in past 4 weeks question and no shading on the manikin. The demographic characteristics of those who provided complete pain data and those who did not were compared using χ^2^ and one way analysis of variance (ANOVA).

Latent class analysis (LCA) was used to identify a substantive number of clusters with different combinations of 16 pain sites. LCA takes into account not only the number of sites of pain but also the location of the sites. In the same cluster, people were assumed to have similar combinations of pain sites and similar numbers of pain sites.^30^ LCA models were fitted successively, starting with a one-cluster model and adding another cluster for each successive model. Model fit was assessed by Bayesian information criterion (BIC), the Lo, Mendell and Rubin (LMR) adjusted likelihood test^31^ and manual inspection of bivariate residuals.^32^ ^33^ The smallest BIC value and a low proportion of bivariate residuals with values above 1.96 indicate good model fit, while the LMR test assesses whether adding one further cluster significantly improves the model fit. Cluster distinction was measured using cluster average posterior probabilities (AvePPs), where a value of above 0.7 represents clear separation.^34^ We aimed to identify a simple but distinct classification. Accordingly, if statistical fit indices suggested complex models with diminutive improvement, and models with few clusters gave similar levels of cluster distinction, we would choose the model with the fewer number of clusters. For each cluster in the chosen LCA model, item conditional probabilities of pain sites give the probabilities that a participant in that cluster reported pain at specific site(s). These probabilities were examined to determine the cluster-specific characteristics of pain sites, with clusters labelled according to an arbitrary cut-off of probability of pain at each site of ≥0.5.

The distribution of pain clusters according to demographic factors and other health factors are presented as frequencies and percentages, or mean and standard deviation (SD), together with the number of participants with complete data for each variable of interest. χ^2^ and ANOVA tests examined the strength of the associations between the pain clusters and all other measures. The median number of pain sites (and inter-quartile range (IQR)) within each pain cluster was calculated. The association of pain clusters with health factors overall, adjusted for age, sex, BMI and number of morbidities, was analysed using multinomial logistic regression models, presented as relative risk ratios (RRR) with 95% confidence intervals (CIs). Participants with complete data for all variables were included in the regression analyses. Uncertainty of cluster membership was taken into account by including each participant’s posterior probability of being in the assigned cluster as a weighted measure.

All LCA models were estimated with 1000 randomly starting values via Mplus V.7.0. Statistical analysis was performed using STATA V.12.1 and IBM SPSS Statistics 20.0.0 (2011).

Multiple imputation was applied to assess the impact of missing data on the results of the multinomial logistic regression. Imputations were performed using the -ice- package in STATA statistical software (release 12.1; StataCorp LP),^35^ and using the guidelines outlined.^36^

## Results

From the eligible study population, 13 986 people completed and returned NorStOP questionnaires, giving an adjusted response of 70.6% (figure 1). Of those who completed the questionnaires, 1578 did not provide complete pain data (275 answered ‘yes’ to pain in past 4 weeks but did not shade on the manikin; 77 answered ‘no’ to pain in past 4 weeks but shaded pain on the manikin; 1226 did not answer pain in past 4 weeks question). Participants with complete pain data were more likely to be male (44.3% vs 41.6%) and younger (mean (SD) age 66.0 (10.2) years vs 70.3 (10.4) years) than those who did not provide complete pain data. The 12 408 (88.7%) participants providing complete pain data formed the final sample for analysis in the current study. Of these, 10 538 (85%) had complete data on all other measures.

A total of 6910 (55.7%) participants were female, and the mean (SD) age was 66.0 (10.2) years, range 50–99 years. A total of 8890 (71.6%) participants reported pain. 1085 (8.7%) participants reported pain at a single site and 7805 (62.9%) at two or more sites. The median (IQR) number of pain sites was 3 (5). 5047 (73.0%) females reported at least one pain site compared with 3843 (69.9%) males (p<0.001). There was no overall age difference in the reporting of pain between the sexes (mean (SD) age 66.7 (10.6) years in females vs 65.2 (9.7) years in males).

Positive ABS scores were defined as mild (1.00–12.65), moderate (12.66–24.61), severe (24.62–47.12) and very severe (47.13–100.00) cognitive complaint, with each category representing 25% of participants with a score between 1 and 100 on the ABS.

There was no statistically optimal model based on BIC and LMR tests. However, the magnitude of decrease in the log-likelihood and proportion of bivariate residuals over 1.96 lessened after extracting ≥6 clusters which suggested that extracting >5 clusters did not improve model fit substantively. When compared to the 5-cluster model, participants in the 4-cluster model generally displayed higher posterior probabilities of belonging to their assigned clusters (AvePPs ranging from 0.86 to 0.95 across the four clusters). Therefore, the 4-cluster model was preferred (table 1).

**Table 1:** Fit indices of the latent class analysis models of 16 pain sites

	Log-likelihood	% reduction in LL from a previous model	BIC	LMR p value	% of significant* bivariate residuals
1 cluster	−98 807	–	197 764	–	100
2 clusters	−85 254	14	170 819	<0.001	97.5
3 clusters	−81 974	4	164 419	<0.001	64.2
4 clusters	−79 633	3	159 898	<0.001	54.8
5 clusters	−78 181	2	157 155	<0.001	45.8
6 clusters	−77 499	1	155 950	<0.001	40
7 clusters	−76 978	1	155 068	<0.001	40

*Values greater than 1.96 indicate significance.

BIC, Bayesian Information Criterion; LL, Log-likelihood; LMR, Lo, Mendell and Rubin adjusted likelihood test.

Table 2 shows the cluster-specific probability for having pain at each site, given membership of that cluster, for each of the four clusters. The first cluster (n=4537; 36.6%) had very low probabilities of pain at any site (<0.09), a median (IQR) number of pain sites of 0 (0) (ie, a no pain or low count of pain sites cluster) and was labelled *Cluster 1*. The second cluster (n=3904; 31.5%) had no probability of pain in the lower back, low probabilities of pain in any particular other site apart from the knee (0.56), and a median (IQR) number of pain sites of 3 (3); this medium count of pain sites (with no back pain) cluster*,* was labelled *Cluster 2*. The third cluster (n=2219; 17.9%) had high probabilities (0.72–0.90) of pain in the spine, lower back and buttock, with low probabilities of pain in any particular other site, and a median (IQR) number of pain sites of 5 (3); this medium count of pain sites (with back pain) cluster, was labelled *Cluster 3*. The fourth cluster (n=1748; 14.1%) had high probabilities (0.55–0.91) of pain at 12 of the 16 pain sites, a median (IQR) number of pain sites of 10 (3) (ie, a high count of pain sites) and was labelled *Cluster 4*. Average posterior probabilities were at least 86% for each cluster, indicating a low chance of misclassification.

**Table 2:** Cluster-specific characteristics: the probabilities of reporting pain at each pain site in each cluster

	Cluster 1	Cluster 2	Cluster 3	Cluster 4
Total number of participants (%)	4537 (36.6)	3904 (31.5)	2219 (17.9)	1748 (14.1)
Head	0.018	0.109	0.109	0.375
Neck	0.001	0.072	0.034	0.290
Chest	0.003	0.125	0.060	0.347
Abdomen	0.022	0.120	0.119	0.356
Shoulder	0.021	0.439	0.322	**0.894**
Elbow	0.001	0.203	0.055	**0.583**
Forearm	0.000	0.191	0.046	**0.651**
Hand	0.030	0.317	0.159	**0.799**
Spine	0.002	0.222	**0.900**	**0.905**
Upper back	0.000	0.173	0.267	**0.613**
Lower back	0.000	0.000	**0.717**	**0.548**
Buttock	0.031	0.162	**0.861**	**0.794**
Knee	0.087	**0.561**	0.405	**0.875**
Calf and shin	0.004	0.321	0.156	**0.592**
Foot	0.038	0.324	0.193	**0.724**
Thigh	0.028	0.270	0.281	**0.630**
AvePP (95% CI)	0.951 (0.948 to 0.953)	0.862 (0.857 to 0.868)	0.929 (0.924 to 0.934)	0.912 (0.905 to 0.918)

Cluster-specific probabilities ≥0.5 are shown in bold.

AvePP, average posterior probability for participants having membership of their assigned cluster.

Age was associated with cluster membership (table 3). Younger (50–64 years) participants were more likely to be in Cluster 4, and those aged 65+ years were more likely to be in Cluster 2. Sex was also associated with cluster membership; females were more likely than males to be in Cluster 4 (ie, to have a high count of pain sites). Mean values for other characteristics (BMI, anxiety, depression, morbidities, cognitive complaint and sleep problems on most nights) were significantly higher in Cluster 4 (p<0.001) than the two medium count clusters, in which the values for these other characteristics were generally similar (table 3).

**Table 3:** Description of pain clusters according to age group, sex, BMI, morbidities and health factors

	Pain cluster (n=12 408)				p Value
	Cluster 1* (n=4537)	Cluster 2† (n=3904)	Cluster 3‡ (n=2219)	Cluster 4§ (n=1748)	
*Age group n(%)*					
50–64	2162 (47.7)	1811 (46.4)	1100 (49.6)	903 (51.7)	<0.001
65+	2375 (52.3)	2093 (53.6)	1119 (50.4)	845 (48.3)	
*Sex n(%)*					
Male	2137 (47.1)	1741 (44.6)	998 (45.0)	622 (35.6)	<0.001
Female	2400 (52.9)	2163 (55.4)	1221 (55.0)	1126 (64.4)	
*BMI*					
Mean (±SD)	25.85 (4.14)	26.77 (4.68)	26.59 (4.46)	27.86 (5.70)	<0.001
*Morbidities n(%)*					
Chest problems	647 (14.3)	867 (22.2)	466 (21.0)	608 (34.8)	<0.001
Heart problems	644 (14.2)	698 (17.9)	414 (18.7)	463 (26.5)	<0.001
Deafness	651 (14.3)	781 (20.0)	442 (19.9)	403 (23.1)	<0.001
Eyesight problems	799 (17.6)	840 (21.5)	500 (22.5)	520 (29.7)	<0.001
Raised blood pressure	1385 (30.5)	1352 (34.6)	729 (32.9)	714 (40.8)	<0.001
Diabetes	371 (8.2)	321 (8.2)	171 (7.7)	198 (11.3)	<0.001
*Anxiety*					
Mean (±SD)	5.38 (3.75)	6.99 (4.05)	7.27 (4.19)	9.29 (4.47)	<0.001
*Depression*					
Mean (±SD)	3.59 (3.27)	4.95 (3.56)	5.16 (3.59)	7.30 (3.94)	<0.001
*Cognitive complaint n(%)*					
None	2947 (68.1)	1879 (51.0)	1105 (53.2)	467 (28.6)	<0.001
Mild	448 (10.4)	430 (11.7)	228 (11.0)	152 (9.3)	
Moderate	361 (8.3)	468 (12.7)	246 (11.8)	302 (18.5)	
Severe	315 (7.3)	458 (12.4)	252 (12.1)	318 (19.5)	
Very severe	256 (5.9)	447 (12.1)	246 (11.8)	393 (24.1)	
*Sleep*					
Trouble falling asleep *n(%)*					
Not at all	2223 (50.2)	1433 (37.8)	748 (34.5)	354 (20.6)	<0.001
On some nights	1897 (42.9)	1846 (48.6)	1040 (48.0)	823 (47.8)	
On most nights	307 (6.9)	517 (13.6)	380 (17.5)	544 (31.6)	
Wake several times/night *n(%)*					
Not at all	1164 (26.5)	584 (15.3)	301 (13.9)	126 (7.3)	<0.001
On some nights	2366 (53.9)	2021 (53.0)	1097 (50.5)	676 (39.3)	
On most nights	859 (19.6)	1208 (31.7)	773 (35.6)	917 (53.3)	
Trouble staying asleep *n(%)*					
Not at all	2038 (47.2)	1155 (31.0)	599 (28.2)	252 (14.8)	<0.001
On some nights	1796 (41.6)	1831 (49.1)	1002 (47.1)	762 (44.9)	
On most nights	483 (11.2)	744 (19.9)	526 (24.7)	684 (40.3)	
Wake feeling tired *n(%)*					
Not at all	2442 (55.7)	1274 (33.6)	651 (30.1)	227 (13.2)	<0.001
On some nights	1629 (37.2)	1860 (49.1)	1086 (50.2)	797 (46.3)	
On most nights	311 (7.1)	654 (17.3)	426 (19.7)	698 (40.5)	

Numbers of participants: BMI, n=11 863; anxiety, n=12 100; depression, n=12 117; each of 6 morbidities, n=12 408; cognitive complaint, n=11 718; trouble falling asleep, n=12 112; wake several times / night, n=12 092; trouble staying asleep, n=11 872; wake feeling tired, n=12 055.

*Cluster 1 participants had no pain sites or a low count of pain sites.

†Cluster 2 participants had a medium count of pain sites with no back pain.

‡Cluster 3 participants had a medium count of pain sites with back pain.

§Cluster 4 participants had a high count of pain sites.

BMI, body mass index.

The complete case analysis and models based on imputed data yielded very similar coefficients (data available on request from RJL). The results from the complete case analyses are presented here. After adjusting for age, sex, BMI and number of morbidities, all health factors except depression, trouble falling asleep on most nights and trouble staying asleep on most nights were significantly associated with Cluster 2 (table 4). Anxiety, depression, moderate cognitive complaint and three of the four sleep problems were significantly associated with Cluster 3. All health factors, except trouble staying asleep, were significantly associated with Cluster 4. In age-stratified analyses (age 50–64 and age 65+) overall results were similar to those for the complete case analysis, that is, in both age groups, health factors were associated with Clusters 2–4 and the strength of associations with Cluster 4 were stronger than with Clusters 2 and 3 (see online supplementary appendix, tables SA and SB). The main differences between the age groups were: (1) fewer health factors were significantly associated with Cluster 2 in those aged 65+ than age 50–64, (2) depression was significantly associated with Cluster 3 in age 65+ but not in age 50–64, and (3) some levels of cognitive complaint and some sleep problems were more strongly associated with Clusters 2–4 in those aged 50–64 than 65+, particularly waking feeling tired.

**Table 4:** Association of pain clusters with clinically relevant health factors: multinomial multivariate logistic regression models*

Outcome	Total (n=10 538)		
	RRR	95% CI	p Value
*Cluster 1*†			
Reference group	1.00		
*Cluster 2*‡			
Anxiety	1.04	1.02 to 1.06	<0.001
Depression	1.02	1.00 to 1.04	0.125
Cognitive complaint			
None (reference group)	1.00		
Mild	1.30	1.10 to 1.53	0.002
Moderate	1.50	1.26 to 1.78	<0.001
Severe	1.42	1.18 to 1.71	<0.001
Very severe	1.43	1.15 to 1.77	0.001
Trouble falling asleep			
Not at all (reference group)	1.00		
On some nights	1.07	0.94 to 1.21	0.283
On most nights	1.21	0.96 to 1.53	0.110
Wake several times/night			
Not at all (reference group)	1.00		
On some nights	1.18	1.01 to 1.38	0.033
On most nights	1.30	1.06 to 1.59	0.012
Trouble staying asleep			
Not at all (reference group)	1.00		
On some nights	1.19	1.03 to 1.37	0.015
On most nights	1.04	0.82 to 1.31	0.771
Wake feeling tired			
Not at all (reference group)	1.00		
On some nights	1.66	1.47 to 1.88	<0.001
On most nights	2.28	1.83 to 2.82	<0.001
*Cluster 3*§			
Anxiety	1.04	1.02 to 1.06	0.001
Depression	1.04	1.01 to 1.06	0.004
Cognitive complaint			
None (reference group)	1.00		
Mild	1.14	0.93 to 1.38	0.201
Moderate	1.26	1.02 to 1.54	0.028
Severe	1.23	1.00 to 1.53	0.051
Very severe	1.12	0.88 to 1.43	0.365
Trouble falling asleep			
Not at all (reference group)	1.00		
On some nights	1.11	0.96 to 1.28	0.173
On most nights	1.50	1.16 to 1.93	0.002
Wake several times/night			
Not at all (reference group)	1.00		
On some nights	1.18	0.98 to 1.43	0.075
On most nights	1.31	1.03 to 1.67	0.029
Trouble staying asleep			
Not at all (reference group)	1.00		
On some nights	1.17	0.99 to 1.39	0.064
On most nights	1.23	0.94 to 1.61	0.135
Wake feeling tired			
Not at all (reference group)	1.00		
On some nights	1.75	1.52 to 2.02	<0.001
On most nights	2.21	1.74 to 2.82	<0.001
*Cluster 4*¶			
Anxiety	1.04	1.01 to 1.06	0.002
Depression	1.11	1.08 to 1.14	<0.001
Cognitive complaint			
None (reference group)	1.00		
Mild	1.52	1.19 to 1.94	0.001
Moderate	2.60	2.09 to 3.24	<0.001
Severe	2.40	1.91 to 3.01	<0.001
Very severe	2.14	1.66 to 2.76	<0.001
Trouble falling asleep			
Not at all (reference group)	1.00		
On some nights	1.33	1.11 to 1.61	0.003
On most nights	1.93	1.46 to 2.54	<0.001
Wake several times/night			
Not at all (reference group)	1.00		
On some nights	1.18	0.90 to 1.55	0.238
On most nights	1.61	1.17 to 2.21	0.003
Trouble staying asleep			
Not at all (reference group)	1.00		
On some nights	1.26	1.00 to 1.58	0.047
On most nights	1.07	0.78 to 1.47	0.656
Wake feeling tired			
Not at all (reference group)	1.00		
On some nights	2.44	1.99 to 2.99	<0.001
On most nights	4.60	3.50 to 6.05	<0.001

*All data: weighted to the probability of class membership, and adjusted for age, sex, BMI and number of morbidities.

†Cluster 1 participants had no pain sites or a low count of pain sites.

‡Cluster 2 participants had a medium count of pain sites with no back pain.

§Cluster 3 participants had a medium count of pain sites with back pain.

¶Cluster 4 participants had a high count of pain sites.

BMI, body mass index; RRR, relative risk ratio.

## Discussion

These results show for the first time that pain sites form four clusters in a population aged ≥50 years. Two dimensions to the clustering are apparent. The first is number of pain sites: low, medium and high. The significant differences in other characteristics (including emotion, cognition and sleep problems) between these three levels of number of pain sites confirm ‘number of sites’ as a distinctive dimension of pain. The second dimension distinguishes the two medium count clusters by a low or high probability of back pain; this is the only clustering influenced specifically by location, and is notable for the lack of any substantial differences between the ‘no back pain’ and ‘back pain’ medium count clusters with respect to emotion, cognition and sleep. This indicates that location is less important than number of sites as the basis for distinguishing clusters of pain in older people.

A previous study of a population aged ≥16 years used separate LCAs to identify pain sites that co-occurred with each of nine primary pain sites, rather than using a single LCA on all 16 pain sites as in our study, and stratified their data by age 16–44 and ≥45 years.^37^ This makes it difficult to compare the studies and likely accounts for the differing results. Our results contrast with those from an LCA of 10 pain sites in a population of 18–79 year olds which found seven distinct pain classes.^10^ One reason for this discrepancy could be that pain sites were reported in the past 7 days rather than in the past 4 weeks in our study. Another explanation could be age: the mean age was >50 years for only three of the seven pain classes in the previous study (regional lower musculoskeletal pain, widespread musculoskeletal pain and widespread whole body pain).^10^ Their findings support our results that pain sites may form fewer clusters in older people compared with younger adults, and that location of pain sites may become less important with age than number of pain sites.

This study has several strengths. It was a large population study of community-dwelling older adults, 36% of whom were aged ≥70 and 12% of whom were aged ≥80 years. In a previous study of clustering of pain sites, 65–79 year olds represented only 14% of the study population and those aged ≥80 were not included.^10^ Our study included a larger range of pain sites (0–16) than the previous study (0–10), which allowed a more precise estimate of the extent of pain experienced by our population. Pain manikins are widely used in population studies of pain, but can be subject to missing data; however, we have reported previously that the small number of participants who reported pain but did not shade pain on the manikin is unlikely to have influenced the results significantly.^3^ There was a high response to our study, and less than 6% missing data on all measures used in the analysis. The health factors measured in this study are recognised markers of poor health and therefore allow for comparisons with other studies; indeed, our results support the findings from previous studies of populations including older people which have shown that the wider the extent of pain, the worse the reported sleep quality, general psychological health, cognitive decline and cognitive complaint.^7^ ^9^ ^38^ ^39^ We measured several potential confounders of the relationship between pain clusters and health factors, including morbidities such as diabetes, since they are common in those with multisite pain,^40^ and of older age. Although morbidities were self-reported, the agreement between self-reported and medical record data has been shown to be good for diabetes, hypertension and some specific heart problems.^41–43^ Unlike traditional clustering approaches (eg, cluster analysis), LCA groups participants together based on probabilistic modelling using finite mixture distributions; this yields a better set of statistical criteria for examining model fit and has been proven to have lower misclassification rates.^44^ ^45^

There are some limitations in this study. Respondents to the questionnaire were more likely to be female and older than non-respondents. Although this could affect the prevalence of pain, it is unlikely that the main associations between pain clusters and health factors would be affected; indeed, the effect may be offset by those with complete pain data being more likely to be male and younger. The current study was conducted in a more deprived area in terms of health, employment and education, but less deprived in housing and services, than in England overall,^13^ which may limit the generalisability of the findings. The data in this study were cross-sectional, and therefore we cannot specify the direction of the associations. We did not assess physical health outcomes which have been shown to be associated with the extent of pain in older people previously.^1^ ^2^ ^46^ ^47^ A previous LCA of pain sites in 18–79-year olds found that pain clusters were strongly associated with physical functioning,^10^ suggesting that this relationship is a topic for future investigation in older people. The blank manikin in this study may capture acute and chronic pain, which could underestimate the patterns and associations related specifically to chronic pain. However, there is evidence that a blank manikin captures pain of longer duration, more disability and worse severity than a preshaded manikin,^48^ consistent with the characteristics of chronic, rather than acute, pain. Indeed, the assessment of pain duration and severity at each pain site is another potential focus for future study since severity^2^ and duration^49^ have been shown to be linked with extent of pain. Dueñas *et al*^49^ identified two subgroups of people with pain using cluster analysis, one characterised by pain at ≥1 site and generalised pain of longer duration (mean age 58 years, SD 13.7), the other characterised by single site pain either in the back or the head and of shorter duration (mean age 55, SD 16.3). The first ‘worse pain’ subgroup may have some characteristics similar to those of Cluster 4 our study, and would support the hypothesis of longer pain duration being a feature of Cluster 4. However, it is difficult to directly compare the results because of differences in the age range (18 years and over) and fewer number of pain sites measured (6) in the Dueñas study. Lastly, there may be unmeasured confounders in this study; for example, we did not assess pain-relief medication which may have been a potential confounder in the relationship of pain with sleep, anxiety, depression and cognitive complaint.

This study provides further argument in favour of number of pain sites as a more important and useful classification of pain in people aged ≥50 years than a traditional regional pain approach based on location alone.^38^ The observation that people with back pain might usefully be classified, for better assessment of their prognosis and better targeted treatment, into those with and without pain elsewhere, has been proposed before^50^ and is supported by other previous research.^1^ ^2^ ^5^ ^6^ ^47^ We have added to this by showing that, regardless of location, the number of pain sites provides the strongest clustering dimension and link with other markers of health. The core message from our study is that, by ignoring the extent of pain, clinicians and public health bodies are overlooking a potentially highly clinically relevant characteristic. We suggest that the four-cluster classification would be useful in primary care because, currently, one of the reasons for the lack of success in treating single site pain complaints in isolation is that the presence of pain at other sites is ignored.^50^ A simple classification of individual patients with pain, based on the four-cluster pattern, could offer a practical approach to the common problem of older persons with multisite pain who present in primary healthcare with single-site pain. The impact of such a classification in practice would need to be tested in studies which directly assess the outcome of decision-making^51^ based primarily on the number of pain sites. It may also be useful to investigate the hypothesis that the four clusters might correspond to the biological distinction between central and peripheral pain and may overlap with, but provide more distinctive classifications than, those based on location alone.

In conclusion, this unique classification of pain sites indicates that pain forms four clusters in a general population aged ≥50 years. Clusters are shaped primarily by number of pain sites (low, medium and high) but also by location (presence or absence of back pain) within the medium cluster. The number of clusters is lower than identified in previous research which means that these four clusters have potential for further investigation as a practical basis for improved clinical assessment and treatment of multisite pain in older primary care patients. This study provides strong evidence in favour of developing and testing simple classifications of pain in older people in general practice based primarily on the number of pain sites rather than a system driven by location alone.

## References

[1] Keenan AM, Tennant A, Fear J Impact of multiple joint problems on daily living tasks in people in the community over age fifty-five. Arthritis Rheum 2006;15:757–64. 10.1002/art.22239 17013823

[2] Croft P, Jordan K, Jinks C “Pain elsewhere” and the impact of knee pain in older people. Arthritis Rheum 2005;52:2350–4. 10.1002/art.21218 16052574

[3] Lacey RJ, Belcher J, Rathod T Pain at multiple body sites and health-related quality of life in older adults: results from the North Staffordshire Osteoarthritis Project. Rheumatology (Oxford) 2014;53:2071–9. 10.1093/rheumatology/keu240 24925881 PMC4202023

[4] Eggermont LH, Bean JF, Guralnik JM Comparing pain severity versus pain location in the MOBILIZE Boston study: chronic pain and lower extremity function. J Gerontol A Biol Sci Med Sci 2009;64:763–70. 10.1093/gerona/glp016 19228782 PMC2691797

[5] Carnes D, Parsons S, Ashby D Chronic musculoskeletal pain rarely presents in a single body site: results from a UK population study. Rheumatology (Oxford) 2007;46:1168–70. 10.1093/rheumatology/kem118 17488750

[6] Jordan KP, Kadam UT, Hayward R Annual consultation prevalence of regional musculoskeletal problems in primary care: an observational study. BMC Musculoskelet Disord 2010;11:144 10.1186/1471-2474-11-144 20598124 PMC2903510

[7] Chen Q, Hayman LL, Shmerling RH Characteristics of chronic pain associated with sleep difficulty in older adults: the Maintenance of Balance, Independent Living, Intellect, and Zest in the Elderly (MOBILIZE) Boston study. J Am Geriatr Soc 2011;59:1385–92. 10.1111/j.1532-5415.2011.03544.x 21806564 PMC3307096

[8] Leveille SG, Jones RN, Kiely DK Chronic musculoskeletal pain and the occurrence of falls in an older population. JAMA 2009;302:2214–21. 10.1001/jama.2009.1738 19934422 PMC2927855

[9] Westoby CJ, Mallen CD, Thomas E Cognitive complaints in a general population of older adults: prevalence, association with pain and the influence of concurrent affective disorders. Eur J Pain 2009;13:970–6. 10.1016/j.ejpain.2008.11.011 19110455

[10] Schmidt CO, Baumeister SE Simple patterns behind complex spatial pain reporting? Assessing a classification of multisite pain reporting in the general population. Pain 2007;133:174–82. 10.1016/j.pain.2007.04.022 17570587

[11] Thomas E, Peat G, Harris L The prevalence of pain and pain interference in a general population of older adults: cross-sectional findings from the North Staffordshire Osteoarthritis Project (NorStOP). Pain 2004;110:361–8. 10.1016/j.pain.2004.04.017 15275787

[12] Thomas E, Wilkie R, Peat G The North Staffordshire Osteoarthritis Project—NorStOP: prospective, 3-year study of the epidemiology and management of clinical osteoarthritis in a general population of older adults. BMC Musculoskelet Disord 2004;5:2 10.1186/1471-2474-5-2 14718062 PMC324560

[13] Jordan KP, Thomas E, Peat G Social risks for disabling pain in older people: a prospective study of individual and area characteristics. Pain 2008;137:652–61. 10.1016/j.pain.2008.02.030 18434022

[14] McBeth J, Lacey RJ, Wilkie R Predictors of new-onset widespread pain in older adults: results from a population-based prospective cohort study in the UK. Arthritis Rheumatol 2014;66:757–67. 10.1002/art.38284 24574238 PMC4163719

[15] Bowling A Research Methods in Health. 3rd edn Open University Press, 2009.

[16] Lacey RJ, Lewis M, Jordan K Interrater reliability of scoring of pain drawings in a self-report health survey. Spine 2005;30: E455–8. 10.1097/01.brs.0000174274.38485.ee 16103839

[17] Southerst D, Cote P, Stupar M The reliability of body pain diagrams in the quantitative measurement of pain distribution and location in patients with musculoskeletal pain: a systematic review. J Manipulative Physiol Ther 2013;36:450–9. 10.1016/j.jmpt.2013.05.021 23845196

[18] van den Hoven LH, Gorter KJ, Picavet HS Measuring musculoskeletal pain by questionnaires: the manikin versus written questions. Eur J Pain 2010;14:335–8. 10.1016/j.ejpain.2009.06.002 19699125

[19] Zigmond AS, Snaith RP The hospital anxiety and depression scale. Acta Psychiatr Scand 1983;67:361–70. 10.1111/j.1600-0447.1983.tb09716.x 6880820

[20] Bjelland I, Dahl AA, Haug TT The validity of the Hospital Anxiety and Depression Scale. An updated literature review. J Psychosom Res 2002;52:69–77. 10.1016/S0022-3999(01)00296-3 11832252

[21] Spinhoven P, Ormel J, Sloekers PP A validation study of the Hospital Anxiety and Depression Scale (HADS) in different groups of Dutch subjects. Psychol Med 1997;27:363–70. 10.1017/S0033291796004382 9089829

[22] Bergner M, Bobbitt RA, Carter WB The Sickness Impact Profile: development and final revision of a health status measure. Med Care 1981;19:787–805. 10.1097/00005650-198108000-00001 7278416

[23] Busija L, Pausenberger E, Haines TP Adult measures of general health and health-related quality of life: Medical Outcomes Study Short Form 36-Item (SF-36) and Short Form 12-Item (SF-12) Health Surveys, Nottingham Health Profile (NHP), Sickness Impact Profile (SIP), Medical Outcomes Study Short Form 6D (SF-6D), Health Utilities Index Mark 3 (HUI3), Quality of Well-Being Scale (QWB), and Assessment of Quality of Life (AQoL). Arthritis Care Res (Hoboken) 2011;63(Suppl 11):S383–412. 10.1002/acr.20541 22588759

[24] Kessler S, Jaeckel W, Cziske R Assessing health in musculoskeletal disorders—the appropriateness of a German version of the Sickness Impact Profile. Rheumatol Int 1997;17:119–25. 10.1007/s002960050020 9352607

[25] Jenkins CD, Stanton BA, Niemcryk SJ A scale for the estimation of sleep problems in clinical research. J Clin Epidemiol 1988;41:313–21. 10.1016/0895-4356(88)90138-2 3351539

[26] Boardman HF, Thomas E, Millson DS Psychological, sleep, lifestyle, and comorbid associations with headache. Headache 2005;45:657–69. 10.1111/j.1526-4610.2005.05133.x 15953298

[27] Morphy H, Dunn KM, Lewis M Epidemiology of insomnia: a longitudinal study in a UK population. Sleep 2007;30:274–80. 17425223

[28] Campbell P, Tang N, McBeth J The role of sleep problems in the development of depression in those with persistent pain: a prospective cohort study. Sleep 2013;36:1693–8. 10.5665/sleep.3130 24179303 PMC3792387

[29] Jordan KP, Sim J, Moore A Distinctiveness of long-term pain that does not interfere with life: an observational cohort study. Eur J Pain 2012;16:1185–94. 10.1002/j.1532-2149.2012.00118.x 22887341 PMC3443361

[30] Magidson J, Vermunt J Latent class models. In: Kaplan D, ed. The Sage handbook of quantitative methodology for the social sciences. Thousand Oakes: Sage Publications, 2004:175–98.

[31] Nylund KL, Asparouhov T, Muthén BO Deciding on the number of classes in latent class analysis and growth mixture modeling: a Monte Carlo simulation study. Struct Equ Model 2007;14:535–69. 10.1080/10705510701575396

[32] Muthén B, Asparouhov T Growth mixture modeling: analysis with non-gaussian random effects. In: Fitzmaurice G, Davidian M, Verbeke G, eds Longitudinal data analysis. Boca Raton: Chapman & Hall/CRC Press, 2009:143–65.

[33] Muthén LK, Muthén BO Mplus user's guide, 7th Edition. Los Angeles, CA: Muthén & Muthén, 1998–2012.

[34] Clark DB, Jones BL, Wood DS Substance use disorder trajectory classes: diachronic integration of onset age, severity, and course. Addict Behav 2006;31:995–1009. 10.1016/j.addbeh.2006.03.016 16675151

[35] Royston P, Carlin JB, White IR Multiple imputation of missing values: new features for mim. Stata J 2009;9:252–64.

[36] White IR, Royston P, Wood AM Multiple imputation using chained equations: issues and guidance for practice. Stat Med 2011;30:377–99. 10.1002/sim.4067 21225900

[37] Hartvigsen J, Davidsen M, Hestbaek L Patterns of musculoskeletal pain in the population: a latent class analysis using a nationally representative interviewer-based survey of 4817 Danes. Eur J Pain 2013;17:452–60. 10.1002/j.1532-2149.2012.00225.x 23042697

[38] Kamaleri Y, Natvig B, Ihlebaek CM Number of pain sites is associated with demographic, lifestyle, and health-related factors in the general population. Eur J Pain 2008;12:742–8. 10.1016/j.ejpain.2007.11.005 18160318

[39] Lee DM, Pendleton N, Tajar A Chronic widespread pain is associated with slower cognitive processing speed in middle-aged and older European men. Pain 2010;151:30–6. 10.1016/j.pain.2010.04.024 20646831

[40] Leveille SG, Bean J, Ngo L The pathway from musculoskeletal pain to mobility difficulty in older disabled women. Pain 2007;128:69–77. 10.1016/j.pain.2006.08.031 17055167 PMC2555988

[41] Okura Y, Urban LH, Mahoney DW Agreement between self-report questionnaires and medical record data was substantial for diabetes, hypertension, myocardial infarction and stroke but not for heart failure. J Clin Epidemiol 2004;57:1096–103. 10.1016/j.jclinepi.2004.04.005 15528061

[42] Simpson CF, Boyd CM, Carlson MC Agreement between self-report of disease diagnoses and medical record validation in disabled older women: factors that modify agreement. J Am Geriatr Soc 2004;52:123–7. 10.1111/j.1532-5415.2004.52021.x 14687326

[43] Barber J, Muller S, Whitehurst T Measuring morbidity: self-report or health care records? Fam Pract 2010;27:25–30. 10.1093/fampra/cmp098 20019091

[44] Magidson J, Vermunt J Latent class models for clustering: a comparison with K-means. Can J Mark Res 2002;20:37–44.

[45] DiStefano C, Kamphaus RW Investigating subtypes of child development. A comparison of cluster analysis and latent class cluster analysis in typology creation. Educ Psychol Meas 2006;66:778–94. 10.1177/0013164405284033

[46] Mottram S, Peat G, Thomas E Patterns of pain and mobility limitation in older people: cross-sectional findings from a population survey of 18,497 adults aged 50 years and over. Qual Life Res 2008;17:529–39. 10.1007/s11136-008-9324-7 18365768

[47] Natvig B, Ihlebaek C, Grotle M Neck pain is often a part of widespread pain and is associated with reduced functioning. Spine 2010;1:E1285–9. 10.1097/BRS.0b013e3181e38e73 20938391

[48] Lacey RJ, Lewis M, Sim J Presentation of pain drawings in questionnaire surveys: influence on prevalence of neck and upper limb pain in the community. Pain 2003;105:293–301. 10.1016/S0304-3959(03)00244-6 14499447

[49] Dueñas M, Salazar A, Ojeda B A nationwide study of chronic pain prevalence in the general Spanish population: identifying clinical subgroups through cluster analysis. Pain Med 2015;16:811–22. 10.1111/pme.12640 25530229

[50] Hartvigsen J, Natvig B, Ferreira M Is it all about a pain in the back? Best Pract Res Clin Rheumatol 2013;27:613–23. 10.1016/j.berh.2013.09.008 24315143

[51] Croft P, Altman DG, Deeks JJ The science of clinical practice: disease diagnosis or patient prognosis? Evidence about “what is likely to happen” should shape clinical practice. BMC Med 2015;13:20 10.1186/s12916-014-0265-4 25637245 PMC4311412

